# The Experiences of LGBTQ+ Pre-Service and Qualified Teachers and Their Mental Health: A Systematic Review of International Research

**DOI:** 10.3390/ijerph23010115

**Published:** 2026-01-17

**Authors:** Jonathan Glazzard, Scott Thomas

**Affiliations:** 1 School of Education, University of Hull, Kingston upon Hull HU6 7RX, UK; 2 Independent Researcher, Leicester LE18 1AD, UK

**Keywords:** pre-service teachers, initial teacher education, LGBTQ+

## Abstract

**Highlights:**

**Public health relevance—How does this work relate to a public health issue?**

- LGBTQIA+ teachers are more likely to experience mental ill-health.
- LGBTQIA+ teachers are exposed to additional stressors which are layered on top of general stressors.

**Public health significance—Why is this work of significance to public health?**

- The paper provides practical suggestions which will reduce mental ill-health in LGBTQIA+ teachers and pre-service teachers.
- The paper suggests solutions which, if addressed at a systemic level, will reduce the prevalence of mental ill-health in LGBTQIA+ teachers.

**Public health implications—What are the key implications or messages for practitioners, policy makers and/or researchers in public health?**

- Initial teacher education courses need to address LGBTQIA-related content explicitly.
- The is a need for more research on the experiences of LGBTQIA+ pre-service teachers, including longitudinal studies which study this population over time, including capturing their transitions into teaching and leadership roles.

**Abstract:**

Existing research highlights that Lesbian, Gay, Bisexual, Transgender and Queer (LGBTQ+) teachers are often exposed to additional stressors in schools which adversely affect their mental health. Some mitigate the effects of these stressors by separating their personal and professional identities while others choose to integrate their identities so that they can be authentic, advance social justice in school contexts or be visible and vocal role models. Less is known about the experiences of pre-service teachers who are undertaking teacher preparation programmes. This systematic literature review presents the results of 20 published papers which represent the global experiences of both pre-service teachers and serving teachers. The findings highlight identity management, experiences of discrimination, agency and lack of confidence of teacher educators. Two new frameworks are presented that lay the foundations for embedding LGBTQ+ inclusion and proposed mandatory elements of curricula for initial teacher training. This systematic literature review has been informed by the following research questions RQ1. What are the experiences of LGBTQ+ pre-service teachers? RQ2. How do LGBTQ+ pre-service teachers negotiate their identities? RQ3. How do LGBTQ+ pre-service teachers disrupt hetero/cis-normative cultures in schools? RQ4: How well does the teacher education programme prepare pre-service teachers for teaching LGBTQ+ inclusive education?

## 1. Introduction

The journey of becoming a teacher is a transformative process, often marked by personal growth, pedagogical development, and professional identity formation. For LGBTQ+ pre-service teachers, this journey is further complicated by the need to navigate their sexual orientation and/or gender identity within hetero/cis-normative educational institutions. These individuals often face unique challenges, including discrimination, lack of representation, and limited institutional support. This systematic literature review explores the lived experiences of LGBTQ+ pre-service teachers, examining the barriers they encounter, the coping strategies they employ, and the support systems that can foster inclusive and affirming teacher education environments.

LGBTQ+ teachers are forced to negotiate their personal and professional identities and engage in identity management strategies [1]. They are often marginalised [2,3,4,5], constantly vigilant and invisible [6] and may avoid opportunities for career advancement [7]. As a result of their experiences of discrimination, they may lack confidence in their teaching abilities, have low self-esteem [8] and develop poor mental health. In contrast, some may attempt to overcompensate for their queer identities by investing their efforts into their professional identity [4,9] so that they can excel in their teaching careers.

Despite these challenges, it is also important to recognise that queer teachers are not always positioned as victims. In fact, some choose to disrupt hetero-and cis-normative school cultures [10,11,12,13] through acts of queering. We deliberately use the term ‘queering’ to represent ways in which queer teachers might disrupt the status quo by taking steps to advance inclusion and social justice in their schools. However, deciding to do this does not come without risks and, arguably, being a queer teacher who engages in acts of queering may also heighten their vulnerability.

Some LGBTQ+ teachers choose to openly disclose their personal identities to be visible role models to students and colleagues. Disclosure may not always be a choice, and some may feel pressurised to act as role models [14]. However, visibility is not always straightforward and can result in suspicion [4], parental backlash [15], prejudice and discrimination. Some may be motivated to actively disrupt rigid hetero/cis-normative structures, policies and practices, thus making deliberate attempts to queer school cultures. Disrupting heteronormativity has been given attention in recent research [10,11,12,13] and is worthy of attention, given that heteronormativity continues to manifest itself in subtle ways [16], for example through the bullying of LGBTQ+ youth in schools [16,17]. Queer teachers and their allies may attempt to trouble hegemonic norms of gender and sexuality through the work that they do to advance social justice within their schools. However, visibility and acting as a role model can result in psychological impacts and isolation [18,19] and places LGBTQ+ teachers in a complex, vulnerable position within schools [20,21]. The work needed to be undertaken to queer schools is often assigned to teachers who are LGBTQ+ [22] and arguably this can eventually take its toll [16]. Queer teachers may be accused of pushing forward their own ‘agenda’ [16], they may be viewed as being a risk to children or seen as hypersexual [8,23] due to discourses of child protection and damaging stereotypes.

Hetero/cis-normativity is prevalent in contemporary schools [1]. This is characterised by the use of gendered language, the separation of learners by biological sex, the emphasis on heterosexual sex education and homophobic bullying. Within heteronormative school cultures, heterosexuality is naturalised and dominant and therefore schools are part of the ‘heterosexual matrix’ [24]. According to Neary [25], the ‘desexualised’ space of the school staffroom is ‘embedded with assumptions of heterosexuality’. Schools can therefore be oppressive spaces which privilege heterosexuality [14,26] and police teacher sexuality [27]. LGBTQ+ teachers may not feel able to, or be permitted to talk about their personal lives and relationships due to the moral panic which prevails around integrating sexuality into education [14]. In addition, the assumption that gender is a binary continues to be prevalent in schools [3] through the titles that children must use to address teachers (Mr, Mrs, Miss, Sir) [28] and single sex grouping arrangements in some schools. Within this context, LGBTQ+ teachers often utilise identity management strategies, including partial disclosure, passing off as ‘straight’ or taking active steps to conceal their true identities. Thus, the identities of queer teachers are often ‘identities under-construction’ [1]. They may choose to conceal or perform their queerness in their interactions and relationships with others, but choosing to engage in acts of ‘queering’ does not come without risks. For some, they may decide that the risks are not worth taking and they may choose to conceal their true identities. Meyer [29] identifies concealment as a strategy which is employed due to exposure to proximal stressors. These are internal stressors which arise when individuals with minoritized identities anticipate a negative reaction from others. Proximal stressors can lead to mental ill-health especially when queer teachers develop internalised homo/transphobia. To protect themselves from discrimination, they may avoid disclosure by separating their personal and professional identities. Some may choose to intertwine these two identities due to their determination to advance social justice and challenge hegemonic norms.

Critically evaluating academic articles is useful, although we recognise that this is a non-standard step for a PRISMA-compliant systematic review. Nonetheless, we chose to undertake a critical analysis of four papers to provide us with contextual information prior to undertaking the systematic review. We decided it is important to state that although the analysis in Table 1 is largely positivist, researchers investigating LGBTQIA+ topics or those who utilise queer and critical theory may situate their work within interpretivist or critical paradigms and may challenge our positivist critique. This process ensures that the review is comprehensive, rigorous, and grounded in high-quality evidence.

### 1.1. Key Benefits

The following points outline the key benefits of evaluating an initial four academic articles to inform this systematic literature review.

1. Enhances analytical depth: Evaluating multiple articles allows researchers to compare methodologies, findings, and theoretical frameworks, leading to a more nuanced understanding of the topic [35].
2. Identifies research gaps: Through critical analysis, researchers can pinpoint areas that lack sufficient investigation, helping to shape future research questions [36].
3. Improves methodological rigor: Assessing the strengths and limitations of each article ensures that only robust and reliable studies are included in the review [37].
4. Supports evidence-based conclusions: Synthesizing insights from multiple sources strengthens the validity of conclusions drawn in the literature review [38].

### 1.2. Theoretical Framework

The preparation of LGBTQ+ pre-service teachers is a critical area of inquiry in teacher education. Understanding the theoretical frameworks and concepts that underpin their experiences is essential for developing inclusive pedagogical practices. This critical analysis explores key theories such as queer theory, intersectionality, critical pedagogy, and identity development models, and evaluates their relevance and application in the context of LGBTQ+ pre-service teacher education. In outlining these theories, there are tensions between them which need to be acknowledged. While queer theory dismantles identity categories, intersectionality recognises the existence of these categories and the impact of multiple minoritized identities on individuals. We draw on queer theory in our analysis of the results because the theory highlights the role of queering as a deconstructive practice, and we argue that pre-service teachers can play a critical role in queering the school curriculum and school cultures. We also highlight the ways in which teacher educators can engage in act of queering. We highlight intersectionality as a concept because it is important to recognise that queer pre-service teachers and learners in schools have racial, ethnic, gendered and other identities which may lead to multiple forms of discrimination. We emphasise in our discussion the need for teacher educators to take account of this. We draw specifically on critical pedagogy to highlight the role of school and teacher education curricula in advancing inclusion and social justice.

### 1.3. Queer Theory

The work of Judith Butler [24] has made a significant contribution to queer theory. Queer theory challenges normative assumptions about gender and sexuality, offering a lens through which to deconstruct heteronormativity in education. It emphasizes fluidity, resistance to categorization, and the interrogation of power structures [39]. For LGBTQ+ pre-service teachers, queer theory provides a framework to critique curricula, pedagogical practices, and institutional policies that marginalize non-normative identities. However, its abstract nature can limit practical application in teacher education programmes [40].

### 1.4. Intersectionality

Intersectionality, coined by Crenshaw [41], examines how overlapping identities, such as race, gender, disability, sexuality, and class, interact to produce unique experiences of oppression and privilege. This framework is particularly relevant for LGBTQ+ pre-service teachers of colour, who navigate multiple axes of marginalization. Intersectionality encourages teacher educators to consider the complexity of identity and to design inclusive curricula that reflect diverse lived experiences [39].

### 1.5. Critical Pedagogy

Critical pedagogy, rooted in the work of Paulo Freire [42], advocates for education as a practice of freedom and social justice. It encourages teachers to question dominant ideologies and to empower students through dialogue and critical reflection. For LGBTQ+ pre-service teachers, critical pedagogy offers tools to challenge oppressive practices and to foster inclusive classroom environments [43]. However, its implementation requires institutional support and a commitment to equity at all levels of teacher education.

### 1.6. Identity Development Models

Identity development models, such as Cass’s model of homosexual identity formation [44], provide insights into the psychological processes LGBTQ+ individuals undergo. These models help teacher educators understand the stages of identity disclosure and integration, which can inform supportive practices for LGBTQ+ pre-service teachers. Later models emphasize the non-linear and contextual nature of identity development [45], aligning with the fluidity emphasized in queer theory.

### 1.7. Application in Teacher Education

Integrating these theoretical frameworks into teacher education requires intentional curriculum design, faculty training, and institutional commitment. Programmes must move beyond tokenistic inclusion to embed LGBTQ+ perspectives across teaching, assessment and field experiences. Mentorship, safe spaces, and policy advocacy are essential components of an affirming educational environment [46]. Moreover, teacher educators must engage in ongoing critical reflection to address their own biases and to model inclusive practices.

### 1.8. Challenges and Limitations

Despite the potential of these frameworks, challenges remain. Resistance from conservative stakeholders, lack of faculty expertise, and institutional inertia can hinder progress. Additionally, the theoretical complexity of concepts like queer theory may alienate some teacher educators and pre-service teachers. Balancing theory with practical strategies is crucial for meaningful integration into teacher education [43].

Theoretical frameworks such as queer theory, intersectionality, critical pedagogy, and identity development models offer valuable insights for supporting LGBTQ+ pre-service teachers. A critical analysis of these theories reveals both their transformative potential and the challenges of implementation. To foster inclusive and equitable teacher education, institutions should consider integrating these theoretical perspectives into curricula to support the advancement of social justice.

In this systematic review, we use the term ‘pre-service teachers’ to refer to those teachers who are undertaking a programme of teacher preparation prior to their qualification as teachers. We use ‘initial teacher education’ (ITE) or ‘initial teacher training’ (ITT) interchangeably, although we recognise there are differences between these terms and that there is considerable debate in the sector about the use of the term ‘training’. We refer to ‘placement’ or ‘practicum’ to refer to periods of structured time in schools which is a mandatory part of teacher preparation programmes, and which enables pre-service teachers to practise the skills of teaching. In addition, we generally adopt the acronym LGBTQ+ to represent teachers who are lesbian, gay, bisexual, transgender, queer or those with other minoritized identities. However, in some sections we use the acronym LGBTQIA+ to add intersex (I) and asexual (A) identities when this acronym has been adopted by specific authors in the papers that we reviewed.

### 1.9. Rationale for This Systematic Literature Review

Existing studies tend to focus on the experiences of teachers who are LGBTQ+ and there are limited studies which attend to the experiences of pre-service teachers.

The questions were developed following a review of key documents as noted in Table 1. Thus, this systematic literature review has been informed by the following research questions (RQ):

- RQ1. What are the experiences of LGBTQ+ pre-service and qualified teachers?
- RQ2. How do LGBTQ+ pre-service and qualified teachers negotiate their identities?
- RQ3. How do LGBTQ+ pre-service and qualified teachers disrupt hetero/cis-normative cultures in schools?
- RQ4: How well does the teacher education programme prepare pre-service teachers for teaching LGBTQ+ inclusive education?

## 2. Materials and Methods

### 2.1. Focus of Our Review

The selection of studies within this systematic literature review are dated between 2013 and 2024. The reason for the long date range was to capture all of the recent and seminal research and statistical data, and relevant legislation. Our focus was to capture the experiences of pre-service teachers who identify as LGBTQ+.

### 2.2. Statement of Compliance

We confirm that this systematic literature review has followed the PRISMA 2020 guidelines for conducting reviews. The following sections set out our approach to the review, and fully complies with all sections of the PRISMA guidance. In relation to PRISMA checklist item#24a, we confirm that we have not formally registered this review [47] (Supplementary Materials).

### 2.3. Systematic Literature Review Protocol

This review is based on the Evidence for Policy and Practice Information and Co-ordinating Centre’s method [48] for understanding systematic literature reviews. The first step was for us to complete an analysis of four papers [16,30,32,33]. Our research questions guided us in defining clear inclusion/exclusion criteria that narrowed down our focus on the literature that should be included in the review. Table 2 shows the systematic steps we took, and Table 3 shows the inclusion and exclusion criteria we applied to the literature review. Table 4 shows the key words.

### 2.4. Sources Used

A number of relevant databases were searched including Eric, Education Source Ultimate, Sage Research Methods, and ProQuest. Based on previous literature (shown in Table 1), key terms were identified and subsequently refined using ‘Boolean operators’ (Table 4). Table 5 shows the keyword combination search terms used.

### 2.5. Screening, Evaluation and Approach to Synthesis

**Figure 1:**
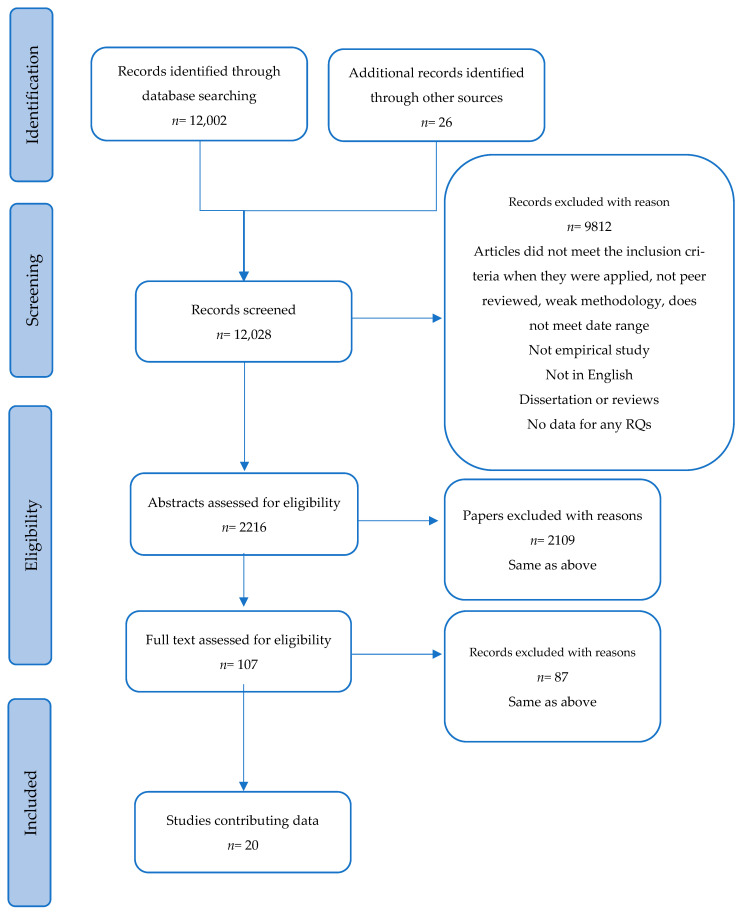
PRISMA flow diagram of study selection.

Screening the studies: Initial keyword search results found 141,380 as shown in Table 6. As combination search terms were used, multiple results for the same articles were found. Following a sift of the search results to remove duplication, the number of individual articles was 12,002 and 26 commissioned reviews and reports. We recognized that this was a significant number of articles to review and so we adopted to use the Preferred Reporting Items for Systematic Reviews and Meta-Analysis (PRISMA) [47], guidelines and checklists to help in the process of screening, as shown in Figure 1. Following our review, 20 studies were identified for inclusion. The studies provide a comprehensive review of global literature which was important for this study. We selected the studies which met the inclusion criteria and utilised the findings to inform the frameworks that we have developed. The PRISMA guidelines (24a) require that we confirm if the review has followed the full registration and protocol. We confirm that registration in line with PRISMA checklist item#24a of the PRISMA guidance was not completed.

Describing and mapping: We first outlined the methodology and key findings from each study, which in turn informed the design of a descriptive map, providing a clear description of each of the individual studies and how they linked to each of the review questions. We split the studies between both authors to complete the descriptive map, and then quality cross-checked each other’s work. An overview of the studies included can be found at Table 7.

Quality and relevance appraisal

- Trustworthiness of results: In-depth analysis of the methodological rigour and competency of design and interpretation of findings.
- Methodological relevance: how appropriate was the study design?
- Weight of evidence: [64] criteria for judging ‘weight of evidence’ was used. The criteria can be seen at Table 8, with the weight of evidence results shown in Table 9.

Synthesising findings: Tables synthesising findings of the studies for each research question were developed. We then used the Narrative Synthesis [48] to capture the main findings from our mapping exercise. Table 10 sets out the synthesized findings in summary that have then been explored in depth throughout this paper.

The analysis of the final 20 papers was undertaken using both a deductive and inductive approach. The broad deductive approach focused on identifying data and themes relevant to the three main research questions informing this review, i.e., (1) What are the experiences of LGBTQ+ pre-service teachers? (2) How do LGBTQ+ pre-service teachers negotiate their identities? (3) How do LGBTQ+ pre-service teachers disrupt hetero/cis-normative cultures in schools? (4) How well does the teacher education programme prepare pre-service teachers for teaching LGBTQ+ inclusive education?

This was followed by a more granular, inductive analysis of each article, resulting in a set of emergent themes under each of the above headings. Given the distinctive nature of the empirical and conceptual studies, a decision was made to analyze these separately using the same approach described above. The first phase of the analysis was deductive and focused on summarizing the findings in relation to each of the three main research questions. The second phase was inductive and involved the identification of the themes that informed the development of the frameworks. To arrive at the themes, we independently analyzed each paper to identify provisional themes. We then independently identified the cross-cutting themes from across the papers. We then met to agree on the final themes. Table 11 shows the search strategy which followed a linear approach. Regular search strategy meetings were held between the authors to discuss any disagreements or anomalies that were identified within the data. The phrases selected were informed by the initial research questions.

## 3. Results

The papers were reviewed in relation to the four research questions:

- RQ1. What are the experiences of LGBTQ+ pre-service and qualified teachers?
- RQ2. How do LGBTQ+ pre-service and qualified teachers negotiate their identities?
- RQ3. How do LGBTQ+ pre-service and qualified teachers disrupt hetero/cis-normative cultures in schools?
- RQ4: How well does the teacher education programme prepare pre-service teachers for teaching LGBTQ+ inclusive education?

Each paper was analysed inductively to identify themes within the paper’s content. Cross-cutting themes were then identified and utilized in the presentation of the findings in this section. The cross-cutting themes were identity; role models; discrimination; queering and agency. These themes were then aligned with the research questions as follows. Table 12 shows the identified themes for each of the research questions. In Section 3 we have cross-referenced our findings to the article numbers that we have used in Table 7. We have used square brackets to cite the article numbers.

### 3.1. RQ1. What Are the Experiences of LGBTQ+ Pre-Service and Qualified Teachers?

#### 3.1.1. Mental Health

Five studies produced findings which highlighted adverse effects on queer pre-service and qualified teachers’ mental health [30,49,58,59,62]. The factors which impacted on mental health varied across the studies. These included the following: queer pre-service teachers experiencing anxiety about their future careers [30]; the psychological pressure of visibility and experiencing double consciousness [16]; psychological distress as a result of direct discrimination [50]; the fear of being visible [60]; and anxiety due to receiving threats about potential damage to their future career [61].

Paper 30 highlighted the anxiety that LGBTQIA+ teachers experience about applying for future jobs in teaching. Some pre-service teachers were actively discouraged by colleagues in schools to disclose their personal identities and warned that if they chose to disclose their identities, this would likely lead to discrimination in the interview process. Some pre-service teachers were worried that their sexuality would influence their experiences of work in the future.

#### 3.1.2. Discrimination

Experiences of discrimination can result in mental ill-health [29] for LGBTQIA+ teachers. Discrimination against queer pre-service teachers and qualified teachers was a theme across the papers. The forms of discrimination vary across the papers but include the following:

- LGBTQ+ teachers being criticised for their dress, being told that they did not belong in the school, made to feel unwelcome and subjected to micro-aggressions [50].
- LGBTQ+ teachers being exposed to discriminatory derogatory language in the form of graffiti [32].
- Pre-service teachers who were LGBTQ+ reported feeling vulnerable due to power structures which operate within schools [50].
- Not feeling able to disclose their personal identities resulted in feelings of isolation [50].

The papers highlighted that discrimination manifests in different ways. For example, in Paper 50 feelings of vulnerability due to power imbalances and fear of disclosure were subtle forms of discrimination which were felt, but largely unspoken. These forms of discrimination result in concealment or identities and internalised homo/transphobia which can impact on their mental health [29]. The more direct forms of discrimination also highlighted in Paper 50 and Paper 32 include direct criticisms and exposure to derogatory language which can also trigger poor mental health [29]. Paper 1 discusses the ‘twin stigma’ experienced by lesbian teachers who experienced discrimination through both sexism and homophobia. This paper also highlights the refusal of school management to label homophobic incidents as homophobic, which erases LGBTQIA+ people’s experiences of discrimination in schools.

#### 3.1.3. Agency

The concept of agency refers to the capacity of individuals to act independently and make their own choices. Limits to agency can affect the capacity of individuals to flourish [65], and result in mental ill-health. In the context of LGBTQ+ pre-service teachers, agency involves the ability to navigate and assert their identities within educational environments that may be heteronormative or exclusionary [28]. Russell [30] highlights the intersection of agency and identity, describing the example of a trainee teacher who realises that their identity, and the performance of that identity has the power to ‘change something’. In understanding the transformative nature of pre-service teachers, and trainee teachers who identify as LGBTQ+, agency is an important aspect noted throughout the various studies [30,32,49,50,51,56]. Given the complexities of the teaching environments in which graduates have entered the profession, we find a connection between their resilience and agency. Notably, resilience is often associated with agency, and we suggest that this is an area that would be worthy of further research [50].

Agency is crucial for LGBTQ+ pre-service teachers as it empowers them to challenge normative assumptions and advocate for inclusive practices. Teacher education programmes that foster agency can help these individuals develop resilience and confidence in their professional identities [58,60,66]. Creating the use of gendered, gender-neutral, and safe spaces also needs to be considered to enable pre-service LGBTQ+ teachers to exercise agency over their path into the profession [60].

Despite the importance of agency, LGBTQ+ pre-service teachers often face systemic barriers such as a lack of inclusive curricula, limited representation, and fear of discrimination. These challenges can hinder their ability to fully engage in their roles as educators and affect their mental well-being [51,57].

### 3.2. RQ2. How Do LGBTQ+ Pre-Service and Qualified Teachers Negotiate Their Identities?

Seven of the studies highlighted the tensions that queer teachers experience in negotiating their personal and professional identities. Studies highlighted that identity management was viewed as important [30], necessary [59] and can lead to denial of identity [49], even when teachers know that having to deny one’s identity is wrong. Studies highlighted that hetero-and cos-normative discourses in school can result in panoptic self-surveillance and in some instances the decision to separate private and public selves was enforced [62] due to school cultures.

‘Passing’ as heterosexual or cisgender is sometimes a considered and deliberate strategy for queer teachers [30], particularly when bodies are not marked by subjective identifiers of queerness. However, this paper also addresses how the silencing of sexuality in school contexts can be indicative of internalised homo/transphobia. The self-policing/self-regulation of identities by teachers was highlighted in some papers [58,59], as well as ‘hyper-engagement’ which occurs when LGBTQIA+ teachers deliberately choose to out themselves [59].

One paper specifically highlighted the influence of school micro-cultures in relation to identity management and negotiation [61]. Aspects of school cultures which can impact on disclosure of identity include the socio-economic context of the school community (social class), religious character of the school, fear of dismissal and fear of being overlooked for promotion. This paper also discusses how discourses of professionalism lead to the silencing of personal identities and ultimately result in a separation of personal and professional identities. In addition, myths that link queerness to paedophilia can also lead to a separation of identities and identity management strategies. Another study [1] also highlighted the conflation of gay identities with paedophilia through a parent questioning whether it was appropriate for a gay teacher to be left alone with children. This illustrates the panopticon of heteronormativity [16] through regimes of power which arguably place queer teacher under greater surveillance than others. One paper [62] emphasised how it is possible for some LGBTQIA+ teachers to ‘blend in’ when they do not embody queerness. Another paper [63] also highlighted how ‘coming out’ in school as an LGBTQIA+ teacher can cast doubt on their fitness to hold authority and lead to a fear of losing credibility.

Concealing one’s identity can result in mental ill-health [29]. The papers highlighted how LGBTQ+ pre-service teachers and qualified teachers make decisions about whether to separate or intertwine their personal and professional identities [16,49,50]. They are aware that within school contexts their personal identities may be contested [16], despite having a positive sense of self. One paper drew on the concept of ‘double consciousness’ [67] to illustrate how queer teachers may be aware of the contested nature of their queer identities while, at the same time, being comfortable with who they are. Some queer teachers may choose to separate their school and private lives [16] and others may be worried about being reduced to a singular ‘queer’ identity [16]. Queer teachers may be worried about parental reactions if their sexual or gender identities are disclosed [49,51] and others may negotiate their sexuality to ‘make it safer for public consumption’ [16]. Others may self-regulate their identities by choosing to remain invisible, by not flaunting their queerness or by appearing to be an ‘acceptable gay’ [16,62].

Although some queer teachers can actively make choices about which parts of their identity to share in school [16], one paper highlighted that separating personal and professional identities is difficult to achieve when queer teachers are directly asked questions about their sexuality [49]. In some cases, being asked directly about one’s personal identity removes the element of personal choice because queer teachers may feel obligated to disclose their identities at this point. Although it may be true that ‘coming out’ within professional contexts can provide personal authenticity, is liberating, creates safe and inclusive cultures in schools [50], the degree to which queer teachers can do this varies greatly [50] and is affected by school contexts. In some schools, coming out as queer can be both tricky and risky and result in discrimination [50]. Russell’s [30] research with pre-service teachers highlights the deliberate use of ‘passing’ as a protective strategy [30]. Passing is when queer pre-service teachers or teachers attempt to ‘pass off’ as straight or cisgender. According to Russell, it is a deliberate and considered strategy which enables queer teachers to embody the heterosexual norm. In some instances, a deliberate attempt to distance the personal and professional selves occurs because the ‘teacher self’ urges queer teachers to be cautious about coming out [30]. In relation to this point, queer teachers may feel that their personal identities are not pertinent to teaching [30] and decisions about ‘outness’ may also be influenced by the religious character of some schools [30].

### 3.3. RQ3. How Do LGBTQ+ Pre-Service and Qualified Teachers Disrupt Hetero/Cis-Normative Cultures in Schools?

#### 3.3.1. Role Models

Cutler, Adams and Jenkins [51] have also highlighted that some queer pre-service teachers are worried about being perceived as having a personal ‘agenda’, a danger to students or being construed as unprofessional [51]. These perceptions may influence the extent to which they feel able to support LGBTQ+ children and young people through being active role models. This paper also highlights how queer pre-service teachers emphasise that LGBTQ+ inclusive practices must be the responsibility of all teachers rather being solely the responsibility of queer teachers.

Henderson’s [49] paper highlights that the role model discourse creates an ‘ethical obligation’ on LGBTQ+ teachers to act as an exception to the desexualised and heteronormative school environment [49]. Acting as a role model is an example of queering as a practice within queer theory. This discourse obligates queer teachers to act as a visible and vocal voice of the LGBTQ+ identity. Queer teachers may choose to be role models because of their own experiences at school, although Henderson states that this can result in them being a ‘lonely flag bearer’ [49]. Some may experience feelings of guilt if they choose not to be a role model [4] and this may force them to adopt the role, rather than it being a genuine choice.

Other studies have highlighted the pressure on LGBTQ+ educators to be ‘out’ role models to take the pressure off other teachers to support LGBTQ+ education [50]. The queer teacher can act as a point of contact for LGBTQ+ students [50] and act as a voice for those who are unable to speak up [50]. Other studies have highlighted the ‘moral obligation’ on queer teachers to ‘come out’ for LGBTQ+ students [30] and the role of a supportive adult in fostering a sense of belonging for others [30]. This sense of obligation can result in exposure to proximal stressors [29] due to anxieties about being exposed to negative reactions from others, thus resulting in mental ill-health for LGBTQ+ teachers.

Johnson’s paper [16] emphasises that the psychological pressures associated with visibility must be balanced with being a role model [16]. Johnson draws on Jeremy Bentham’s 18th century concept of the panopticon which was adopted by Foucault [68] in his own concept of hierarchical observation. According to Foucault [68], power is exercised from innumerable points and is not always hierarchical. It often functions in silence rather than being overt, yet it has remarkable effects in relation to how it regulates people’s thinking and behaviours. Johnson’s draws on this body of scholarship to highlight the role of parents as guardians of heteronormativity. Fear of parental backlash against LGBTQIA+ curricula in schools is also documented elsewhere [15]. Recent examples of this have been evident in the UK where parental protests in opposition to LGBTQ+ education outside of schools in 2019 dominated the media headlines. Queer teachers may be worried about parental reactions, they may worry about saying something illegal or incorrect, teaching content which is viewed as difficult or dangerous, and they may be concerned that others, including parents, will perceive them as pushing forward their own agenda [16]. These examples illustrate the informal networks of power which circulate in schools and which, understandably, may deter LGBTQ+ teachers from being role models. Queer teachers know that they play an important role in reducing or eradicating stigma but, as Johnson [16] argues, they are unconsciously affected by school cultures which perpetuate a view that children need to be protected from the real world. The panopticon of heteronormativity impacts on what they can say in the classroom, and they may worry that they are perceived as pushing forward an agenda to turn children queer [16].

One paper [30] discusses the physical embodiment of queerness which sometimes makes it difficult for queer teachers to hide their personal identities. In cases where queerness is embodied, LGBTQIA+ teachers may not have a choice about being a role model.

#### 3.3.2. Queering

We interpret the term ‘queering’ as representing disrupting the hetero/cis-normative discourses which regulate what teachers can say and do in schools. For us, queering represents a direct challenge to the status quo in that we associate it with thinking differently, doing things differently and advancing social justice.

Eight of the studies referred to queer teachers as ‘disrupters’ of cis- and hetro-normative school cultures, although the forms of disruption and reasons for not being a disrupter varied across the papers. The teachers experienced a tension between keeping their careers and promoting positive change to school cultures [30], suggesting that the teachers viewed acts of disruption as potentially risky to their careers. The psychological impact of being a disrupter was highlighted in one study [16] and this study also highlighted that decisions about whether to be a disrupter were influenced by other people’s perceptions of them as having an agenda. One study highlighted that school context can impact on whether or not to disrupt [51] and another study highlighted that pre-service teachers were aware of the challenges associated with queering the school curriculum due to perceived sensitivity associated with LGBTQIA-related content [56]. Examples of forms of disruption or ‘queering’ across the studies included troubling the gender binary in schools and initiating and encouraging open debate with students [63] and utilising the services of external agencies to support the delivery of LGBTQIA+ related content [1]. Giving students permission to ask questions [1] was also a further example of disruting heter- and cis-normative discourses in schools. One study highlighted that teacher educators lacked the confidence to queer the teacher education curriculum and that teacher educators also under-estimated the importance of LGBTQIA+ within the discussion of inclusive education [55].

One of the published papers refers to LGBTQ+ teachers advocating for gender-neutral bathrooms in schools [50]. These teachers felt able to challenge the ‘heteronormative matrix’ [24] and support, nurture and navigate learning opportunities for all [50]. Another paper highlights examples of queering through teachers using gender inclusive language rather than binary language [51], for example, by using ‘children’ or ‘pupils’ rather than ‘boys’ and ‘girls.’ However, in the context of ITE, one study found that pre-service teachers did not feel confident to queer the hetero/cis-normative cultures in their schools because their teacher education course had not adequately prepared them to do this [53]. Evidence in another paper also drew attention to the fact that pre-service teachers had a good level of awareness that school context influences what they are permitted to teach [53] and therefore influences the extent to which they are able to engage in queering practices. Evidence from other studies also suggests that pre-service teachers recognised the restrictions associated with working in a faith school in relation to how this might influence their capacity to employ queering strategies [51]. Tompkins, Kearnes and Mitton-Kükner (2019) found that queer pre-service teachers are often skilled at reading the school climate and experience a sense of positive affirmation when members of the school leadership team help them to feel accepted [50]. However, another paper [51] highlighted how pre-service teachers were aware that the school context impacted on their ability to interrupt hetero-and cis-normative discourses, including the religious character of some schools, school ethos and parental values. Visible signs of queerness in schools, for example, the rainbow flag, engendered within them a sense of belonging and support from school leaders influenced the extent to which they could be authentic and, arguably, the extent to which they could queer their schools. [50] In this paper, the authors illustrate how queering the teacher education curriculum had resulted in one beginning teacher leading LGBTQ+ professional development for other staff members [50].

Although acts of queering or interruption can be informal, one paper [56] discusses the specific implications for pre-service teachers who are LGBTQIA+. This paper highlights how pre-service teachers are caught in a position between doing what they think is morally right and going against the advice of their school-based teacher educators (sometimes known as mentors), who hold considerable influence. This point is critical because it illustrates how differential power can restrict the agency of LGBTQIA+ pre-service teachers. The paper highlights how pre-service teachers are aware that they need to do a good job to pass their course, gain approval from school-based teacher educators and secure good references for future employment. They have a lot to lose if they do not gain the approval of their mentors and they are acutely aware that their careers are at stake. They feel torn between passing the course, gaining certification and their commitment to the advancement of social justice. They therefore occupy a precarious terrain and may decide to leave their social justice work until they are qualified and have their own classes.

In relation to LGBTQIA+ teachers, one paper [1] highlighted how the interrupting of hetero- and cis-normative cultures may be a deliberate decision, but for others it may be less deliberate. The paper gives an example of a teacher who was undergoing gender reassignment where their students set up a social media group in support of their transition. This was an unexpected act of queering but also an act of queering from the students as well as the staff member.

### 3.4. RQ4: How Well Does the Teacher Education Programme Prepare Pre-Service Teachers for Teaching LGBTQ+ Inclusive Education?

Lack of training for teacher educators in LGBTQIA+ content was highlighted as a theme across several papers [32,54,55]. One paper [53] highlighted the importance of providing pre-service teachers with concrete examples of how to address LGBTQIA+ content and the need for teacher educators to provide modelling in relation to how to teach LGBTQIA+ topics in school.

Making LGBTQ+ content visible in the teacher education curriculum was a significant focus on one of the papers [54]. The findings of this study suggest that queer inclusive teacher education programmes can have a positive effect on the attitudes and knowledge of pre-service teachers. Queering the curriculum within teacher education programmes is an example of queer theory and critical pedagogy in action. The aim of queering is to disrupt and destabilise the heteronormative curriculum. Destabilising norms is a critical aspect of queer theory. In addition, embedding aspects of social justice into the curriculum is a key aspect of critical pedagogy.

Examples of queering the teacher education curriculum that this article cites include:

- The inclusion of historical and contemporary significant LGBTQ+ individuals into the ITE subject curriculum, including Harvey Milk and Eleanor Roosevelt.
- Integrating diverse children’s literature into the ITE curriculum, representing diverse family structures.
- Including guest speakers.

In this paper, Brant, and Willox (2021) [54] argue that LGBTQ+ content does not need to be an ‘add on’ to the existing curriculum and can be integrated into the existing subject curriculum. The authors, in emphasising this, are making an important point given that ITE courses at postgraduate level in some countries (for example, the UK) are short and pre-service teachers spend a significant proportion of that time working directly in schools. The authors adopt the metaphor of a ‘mirrors, windows and sliding doors’ as a way of framing how to develop an LGBTQ+ inclusive curriculum in teacher education programmes. In relation to mirrors, LGBTQ+ pre-service teachers must be able to see themselves reflected in the curriculum. The curriculum should provide a ‘window’ into the lived experiences of others, for example, by reading and hearing other people’s stories and including the voices, perspectives and experiences of LGBTQ+ teachers, children and young people. Finally, the concept of ‘sliding doors’ represents becoming part of the world that is represented, for example, by being transported into a story, using drama techniques or through projects which focus on allyship.

One paper emphasises the need for teacher education courses to address the concept of intersectionality, to challenge binary thinking and to embed relevant theorisation [55]. In addition, there are some critical issues that teacher education must attend to. These are summarised below:

- Some teacher educators do not know what the letters in the LGBTQIA+ acronym represent.
- Some teacher educators do not feel prepared to provide LGBTQ+ inclusive teacher education.
- Teacher educators may lack confidence in integrating LGBTQ+ inclusion into the subject curriculum.
- Some teacher educators consider LGBTQ+ inclusive education to be inappropriate for primary-aged children.

Teacher educators felt that they needed support from ‘experts’ to deliver LGBTQ+ inclusive teacher education.

The concept of intersectionality was poorly understood among teacher educators.

## 4. Discussion

It is important to highlight that there is a legal requirement for teachers in England to remain impartial and therefore they cannot exploit their professional position by expressing biased viewpoints which might be harmful to children. Therefore, if teachers have strong anti-LGBTQIA+ views, they must not disclose these to learners. In addition, teachers in England must maintain political impartiality by not expressing personal biased political viewpoints.

LGBTQ+ individuals entering the teaching profession often navigate complex intersections of identity, pedagogy, and institutional culture. Pre-service teachers, those in training before full certification, face unique challenges and opportunities as they prepare to enter classrooms and schools that may or may not affirm their identities. This systematic literature review has explored the lived experiences of LGBTQ+ pre-service and qualified teachers, focusing on identity development, institutional support, challenges in field placements, and the role of teacher education programmes in fostering inclusive environments. The pressure for LGBTQIA+ teachers to disclose their identities by being visible role models can be potentially risky for some teachers and create queer fatigue. It is important not to underestimate the emotional labour that queer teachers are often forced to experience and school leaders should therefore ensure that teachers who are LGBTQIA+ are adequately supported.

In England, government policy on teacher education in has arguably situated pre-service and qualified teachers within a discourse of performativity in which they are held accountable for the progress of the learners that they teach. In his seminal work, Stephen Ball [69] (p. 216) defines performativity as ‘a technology, a culture and a mode of regulation that employs judgements, comparisons and displays as means of incentive, control, attrition and change…’. Notions of what constitutes ‘effective teaching’ and the ‘good teacher’ are shaped by discourses of performativity which values and rewards educational outputs above a commitment to social justice. It results in ‘inauthentic practice’ [69] (p. 222) which marginalises some of the most vulnerable learners. These are often learners who struggle to achieve narrow, academic performance indicators which are valued within a neoliberal, marketized society. The emphasis on raising academic achievement has meant that social justice work in education often becomes marginalised. Discourses of performativity affect pre-service teachers in various ways. First, government mandated teacher education curricula which fails to address social justice related content limits agency because pre-service teachers may not gain opportunities to research, plan and teach aspects of social justice in schools. Second, agency is restricted because schools prioritise aspects of education which are measured, quantified and therefore valued. Developing learners’ attitudes in relation to inclusion and social justice is therefore often viewed as a luxury within the neoliberal, marketised education system which rewards measurable performance indicators rather than changes in attitudes, values and beliefs. Within this performative context, pre-service teachers may not have opportunities to develop a curriculum for social justice.

Despite this contextual backdrop, Philpott [70] (p. 9) argues that ‘some student teachers are not comfortable with the teacher identity they begin to feel it is necessary to adopt’. Tickle [71] (p. 1) argues that ‘we should not think of induction simply as if novices are to be socialised into some well formulated and accepted practices which exist on the other side’. Re-positioning pre-service teachers as agents of social justice invites exciting possibilities to re-think the purposes of education. If the role of education is to support learners to become well-rounded future citizens, social justice work in school classrooms plays a crucial role in facilitating the development of a more socially just society. We argue that pre-service teachers can play a critically important role in helping to change attitudes, tackle prejudice and promote cultures of respect if they are given opportunities to undertake social justice projects with their learners in classrooms. This important work can be undertaken by all pre-service teachers through the introduction of short, focused immersion placements in schools which provide opportunities for pre-service teachers to design schemes of work which address matters of race, disability, gender, poverty and sexual orientation with primary and secondary-aged students.

Teacher education programmes increasingly recognise the importance of diversity and inclusion. Enhancing the visibility of marginal identities, such as those related to race, ethnicity, gender, sexuality, and disability, can foster a sense of belonging among pre-service teachers and support the development of a robust teacher identity [72]. Visibility initiatives contribute to belonging and identity formation, drawing on intersectionality and inclusive pedagogy frameworks [73,74].

Belonging is a fundamental human need and a critical factor in educational success [75,76]. For pre-service teachers representing minoritized groups, visibility within curricula, faculty representation, and peer networks signals acceptance and value [74]. McKay and Manning [72] highlight that preservice teachers who perceive alignment between their social justice commitments and programme ethos report stronger engagement and resilience. Conversely, invisibility perpetuates feelings of isolation and marginalisation, undermining identity development [77].

Teacher identity is dynamic, multifaceted, and shaped by sociocultural and institutional contexts [78,79]. Visibility of diverse identities within teacher education programmes provides mirrors and models that validate candidates’ lived experiences, enabling dialogical identity construction [80]. McKay [81] demonstrates that arts-based reflective practices, such as collage, allow preservice teachers to integrate personal and professional identities, including aspects of self-care, into their evolving teacher identity.

Intersectionality offers a lens to understand how overlapping social markers—race, gender, sexuality, disability—shape experiences of belonging and exclusion [73]. Inclusive pedagogy moves beyond tokenistic gestures to embed diverse voices in curriculum, assessment, and placement practices [82]. Strategies include diversifying reading lists, creating safe spaces for identity expression, and critically interrogating dominant norms in professional practice [83].

Programmes should prioritise structural and cultural changes that normalise diversity as a professional asset. This includes recruiting faculty from underrepresented groups, embedding critical reflection on identity in coursework, and ensuring placement schools model inclusive cultures. Visibility initiatives must be coupled with meaningful support to avoid tokenism and foster authentic belonging [74]. Increasing the visibility of marginal identities within teacher education programmes is not merely symbolic; it is transformative. It fosters belonging, validates lived experiences, and supports the development of strong, resilient teacher identities equipped to enact socially just pedagogies.

Teacher education programmes must directly engage pre-service teachers with diversity through a rich diet of immersion activities, including opportunities to work directly with children and young people who are culturally and linguistically diverse [84]. These immersion activities provide opportunities for pre-service teachers to engage in queering the school curriculum (queer theory) and for embedding social justice content into it (critical pedagogy). Queer theory is concerned, in part, with ‘queering’ as an action which intentionally examines, interrogates and subverts norms and we argue that a diet of rich immersion activities will enable pre-service teachers to engage in acts of queering the school curriculum. In addition, integrating critical discussions into the classroom about matters related to race, sexuality, gender, social class and disability and the intersections between these identities reflects the principles of critical pedagogy. For some, these immersion experiences will be both uncomfortable and transformative. Pre-service teachers can only deeply understand children’s lives and cultures through rich, concrete experiences which force them to re-evaluate their own assumptions and biases [85]. Research demonstrates that teachers who cannot address adequately the needs of a diverse range of students are at risk of burnout [86]. Teacher education courses must therefore fully prepare them to teach students of all races, ethnicities, abilities, sexualities, and genders so that they are empowered to address the professional challenges that they will face throughout their teaching careers. Embedding social justice matters into teacher education programmes strongly aligns with Paulo Freire’s [42] work on critical pedagogy by engaging pre-service teachers in critical discussions about systemic discrimination. In addition, it enables them to nurture the development of positive attitudes and values in the learners that they are responsible for educating.

We recognise, of course, that implementing social justice-oriented immersion placements might not be straightforward, particularly if schools are reluctant for pre-service teachers to do this work. We also recognise that school contexts also constrain or enable what is possible to achieve and that school leaders may wish to prioritise maximising learners’ achievements rather than transforming their attitudes. Arguably, teachers are trapped in a discourse of performativity [69] which restricts their ability to develop socially just pedagogies in their own classrooms [87] and the same is true for school leaders who have to balance their commitments to social justice against the pressure to raise learners’ achievements. Re-thinking teacher education programmes and school placements and re-positioning pre-service teachers as agents of social justice will require changes to teacher education programmes so that matters of race, disability, sexuality, gender and other aspects of social justice such as poverty are given greater prominence. In addition, mentors who support pre-service teachers during school placements will also require professional development and training so that they are more able to support pre-service teachers in schools. Teacher education programmes must support pre-service teachers to reflect critically and deeply on their beliefs, attitudes and values [88] if they are to have a transformative effect.

**Figure 2:**
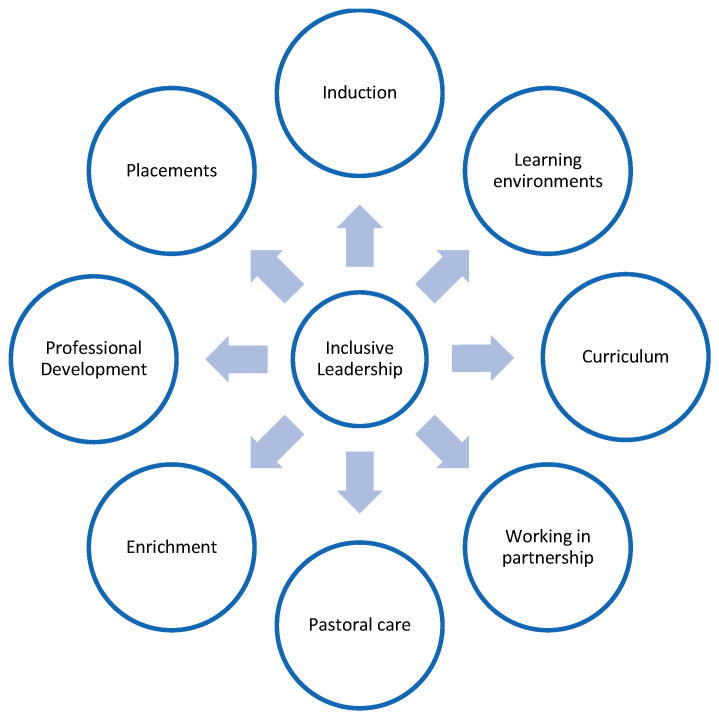
Embedding LGBTQ+ Inclusion in ITE.

**Figure 3:**
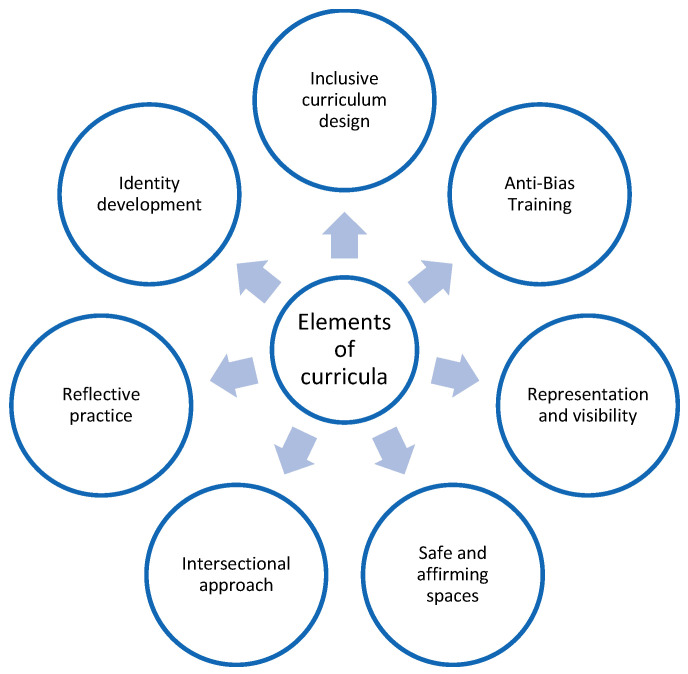
Mandatory elements of ITE curricula.

In addition, teacher educators must be able to ‘interrupt discriminatory and harmful schooling practices’ [88] (p. 67) which perpetuate exclusion. This systematic review has provided clear support for Figure 2 and Figure 3, which have been developed to help enhance the training and support for pre-service teachers. Figure 3, specifically sets out a framework to support LGBTQ+ inclusion in teacher education. Figure 3 sets out six critical elements that, we propose, should be mandatory aspects of ITE curricula. We view these frameworks as valuable models through which teacher educators can queer ITE programmes and through which pre-service teachers can queer curricula in the schools that they are working in. In using the word ‘queer’ as a verb and a praxis, the approach that we have suggested strongly reflects the principles of queer theory [24]. Programmes should provide, for some pre-service teachers, an uncomfortable space to address contested issues of inequality in relation to disability, race, social class, gender and sexuality. Pre-service teachers should be supported to be aware of and confront their own biases, privilege, and prejudices in order to reduce deficit thinking. McKay [89] has highlighted how university teacher education programmes can transform pre-service teachers’ attitudes and values through attending to attitudes, values and beliefs rather than a narrow focus on skills development.

Increasing the visibility of marginal identities on teacher education programmes fosters a sense of belonging and supports the development of a strong teacher identity [72]. However, teacher education providers also need to give attention to the pastoral care of pre-service teachers who are LGBTQIA+ given that they may be exposed to additional stressors and, consequently, some will be concealing their identities, particularly during school placements. Concealment of identities may arise due to internalised stigma [29] and can result in mental ill-health. Some may employ the tactic of ‘passing’ [90] (p. 73) off as heterosexual or ‘covering’ [90] (p. 102) up their identities due to fear of rejection or discrimination. Having access to a supportive nominated mentor during school placements may alleviate some of these tensions, although it is important to ensure that the nominated individual is not responsible for assessing their performance as a teacher.

### 4.1. Identity Development and Self-Disclosure

Identity development for LGBTQ+ pre-service teachers is a deeply personal and often politicized process. Many struggle with whether to disclose their sexual orientation or gender identity during their training, particularly in field placements where school cultures may be conservative or heteronormative. However, given that research [91] highlights that teacher education programmes often lack structured opportunities to learn about LGBTQ+ content, curriculum invisibiliy can result in queer pre-service teachers having to navigate these complexities independently.

The process of becoming an LGBTQ+ teacher is not only about professional identity but also about reconciling personal authenticity with perceived professional expectations. A year-long study of two LGBTQIA+ pre-service teachers revealed that working with mentors in schools who are perceived to hold anti-LGBTQ+ viewpoints can be challenging, leading to emotional labour and stress [92].

### 4.2. Challenges in Teacher Education Programmes

Despite growing awareness of diversity and inclusion, many teacher education programmes still fall short in preparing future educators to address LGBTQ+ issues. According to GLSEN’s comprehensive report, only a minority of teacher educators feel confident integrating LGBTQ+ topics into their curriculum [93]. This lack of preparation contributes to a cycle where LGBTQ+ pre-service teachers feel isolated and unsupported.

Moreover, LGBTQ+ topics are often treated as peripheral rather than integral to diversity education. Research [94] has found that sexual orientation and gender identity were ranked lowest among diversity priorities in teacher training programmes. This marginalization reinforces the invisibility of LGBTQ+ identities in educational discourse and practice.

### 4.3. Field Placements and Institutional Barriers

Field placements are a critical component of teacher training, yet they often present significant barriers for LGBTQ+ pre-service teachers. A case study by Terry et al. [95] documented the experiences of a transgender pre-service teacher navigating multiple school districts in the south-eastern United States. The participant faced discrimination, lack of administrative support, and difficulty securing placements, all of which hindered their professional development.

These experiences are not isolated. Many LGBTQ+ pre-service teachers report feeling unsafe or unwelcome in their placement schools, particularly in rural or conservative areas. The fear of being outed or discriminated against can lead to self-censorship and emotional distress, impacting both teaching performance and mental health.

### 4.4. Support Systems and Resilience

Despite these challenges, LGBTQ+ pre-service teachers often demonstrate remarkable resilience. Support systems, both formal and informal, play a crucial role in fostering this resilience. Faculty mentors, LGBTQ+ student organizations, and affirming peers can provide critical emotional and professional support.

Goldstein-Schultz [96] emphasized the importance of administrative and peer support in shaping positive experiences for LGBTQ+ educators. When teacher education programmes include LGBTQ+-affirming content and provide safe spaces for dialogue, pre-service teachers report higher levels of confidence and preparedness.

### 4.5. The Role of Teacher Educators

Teacher educators are pivotal in shaping inclusive learning environments. However, many lack the training or confidence to address LGBTQ+ issues effectively. According to GLSEN’s [93] report, a significant number of teacher educators had not received professional development related to LGBTQ+ inclusivity. This gap perpetuates a cycle of under preparedness among future teachers.

Programmes that do integrate LGBTQ+ content often do so inconsistently. Some rely on guest speakers or one-off workshops rather than embedding inclusivity throughout the curriculum. This fragmented approach fails to equip pre-service teachers with the tools needed to navigate real-world classroom dynamics involving LGBTQ+ students and colleagues.

### 4.6. Intersectionality and Diverse Experiences

Although it is well documented that queer teachers are more likely to experience discrimination [1,4,49,62,97,98,99,100] there is much less research on the experiences of queer teachers with intersectional identities. It is essential to recognize that LGBTQ+ pre-service and qualified teachers are not homogenous. Their experiences are shaped by intersecting identities, including race, ethnicity, socioeconomic status, and disability. For example, transgender and nonbinary pre-service and qualified teachers often face distinct challenges related to pronoun use, bathroom access, and legal documentation [19,95]. In addition, LGBTQ+ pre-service and qualified teachers of colour may experience compounded marginalization within predominantly white institutions [101], thus resulting in mental ill-health [29]. These intersecting oppressions require nuanced support strategies that go beyond generic diversity training.

### 4.7. Implications for Policy and Practice

Our review of the literature highlights that LGBTQ+ pre-service teachers, teacher education programmes must adopt comprehensive, intersectional approaches to inclusivity [33,95,102,103,104]. This includes:

- Embedding LGBTQ+ topics across all coursework, not just in diversity electives.
- Providing mentorship opportunities with LGBTQ+ educators.
- Creating clear policies that protect against discrimination in field placements.
- Offering professional development for faculty on LGBTQ+ issues.

Policy changes at the institutional and state levels are also necessary to ensure that LGBTQ+ pre-service teachers are not only protected but affirmed in their identities.

Following our review of the literature, we propose a framework to support LGBTQ+ inclusion in teacher education. This is shown in Figure 2.

This section addresses each of the strands in Figure 2. We envisage that the framework will be useful to countries in Europe, Australia, Canada and the US.

The process of inducting pre-service teachers into universities and into school requires careful consideration. During the process of induction, ITE providers and schools should communicate clear messages about their commitments to inclusion and anti-discrimination practice [20]. These commitments should be communicated during university open days, taster events and during the first few days on campus. When pre-service teachers undertake each school experience, mentors in school should also communicate clear messages about their commitments to inclusive values and practices.

Learning environments both in schools and universities should include clear messages about anti-discrimination and ITE providers and schools should ensure that library spaces also include books which represent diverse identities. The learning environment was highlighted by Stones and Glazzard [28] as an important aspect that leaders should consider carefully. LGBTQ+ pre-service teachers should feel comfortable in the learning environment, with support for them to effectively navigate and assert their identities within educational environments that may be heteronormative or exclusionary [28].

LGBTQ+ identities and experiences should be visible across the ITE curriculum within subjects. In addition, the ITE curriculum should address the concept of allyship and the concept of intersectionality. The ITE curriculum should also support pre-service teachers to design schemes of work and lessons which help children to learn about LGBTQ+-related matters so that all pre-service teachers are confident in planning units of work in classrooms. Working in partnership with pre-service teachers is a powerful way of giving them agency. Pre-service teachers can be encouraged to become LGBTQ+ champions or ambassadors and in this role, they can work with ITE subject leaders to support them in integrating LGBTQ+ content into the subject curriculum. Pre-service teachers can also undertake training to become LGBTQ+ allies and ITE providers can shape this role. Partnership also includes collaborations with LGBTQ+ organisations which can work with ITE staff to enhance the ITE curriculum.

We have added pastoral care to the framework to remind ITE providers that LGBTQ+ pre-service teachers might need additional support, given the association between LGBTQ+ and mental health. ITE providers can provide additional support to LGBTQ+ pre-service teachers by discussing how to negotiate school contexts, how to address discrimination and how to address specific questions from children in schools about their sexuality or gender identity/expression. Philpott [70] has highlighted the importance of providing effective pastoral care to pre-service teachers. We suggest that this should be an ongoing support that is not abandoned at the point of graduation.

Curriculum enrichment opportunities might take various forms, but are likely to include visiting speakers, visits to the galleries to view queer exhibitions, visits to the theatre or cinema to watch queer plays and films and visits to schools to learn about how teachers have addressed LGBTQ+ with children. Unwin, Starcevich, Lembo, and Dobson [33], provide practical recommendations for teacher educators and institutions, such as queering the curriculum and creating safe spaces for LGBTQIA+ staff, which underscores the importance of delivering a varied, and culturally inclusive curriculum.

High quality professional development is an important aspect of the framework, and this strand addresses professional development for ITE staff and school-based mentors who are responsible for supporting pre-service teachers in school. All staff, irrespective of their role, need to understand the legal frameworks in which they are working and the implications of these frameworks for their own practice. All staff need to understand how to support pre-service teachers with identities that lie outside the gender binary and those who are transgender. Russell [30] found that the application of borderland discourse theory provides a robust framework which underscores the importance of integrating personal identity into professional development.

The framework includes placements as a distinct strand because pre-service teachers typically spend a large amount of time undertaking periods of practicum in schools. We think that pre-service teachers will benefit from immersion placements in which they undertake short placements, in addition to their longer block placements, in which they focus on designing and teaching lessons which develop children’s knowledge of LGBTQ+ experiences and identities. Pre-service teachers should have opportunities to reflect on these when they return to the university. An important aspect of pre-service teacher education is the visibility of marginal identities on teacher education programmes, which fosters a sense of belonging and supports the development of a strong teacher identity [72]. Terry et al. [95] completed a case study on the experiences of a transgender pre-service teacher in the south-eastern United States, and provide supportive data that placements are an essential element of a pre-service teachers initial training.

Leadership is the central strand which draws together all aspects of the framework. It requires ITE providers to commit to, and articulate, a set of inclusive values which are underpinned by policies which lead to LGBTQ+ inclusive practice at the delivery level [5]. ITE providers. The leadership strand requires providers to finally commit to funding staff development and mentor training [5]. In addition, it requires ITE providers to evaluate their own progress in relation to each of the strands of the framework and to regularly identify priorities for improvement. Therefore, the framework should be underpinned by robust quality assurance processes. It is important for leaders to balance theory with practical strategies to enable meaningful integration into teacher education [43].

Embedding LGBTQ+ inclusion in pre-service teacher education is essential for fostering equitable, affirming, and safe learning environments. We suggest that the curricula used to educate pre-service teachers should include seven specific elements; inclusive curriculum design; anti-bias training; representation and visibility; safe and affirming spaces; intersectional approach, reflective practice and identity development. Figure 3 shows the seven elements that we suggest should be mandatory within the pre-service teacher training curricula.

### 4.8. Best Practices

1. Inclusive Curriculum Design: ITE providers should integrate LGBTQ+ topics across subjects, not just during designated months. The curriculum should reflect diverse identities and experiences [102].
2. Anti-Bias Training: ITE providers should provide training on heteronormativity, homophobia, and transphobia to challenge biases and promote inclusive pedagogy [104].
3. Representation and Visibility: Include LGBTQ+ educators, authors, and historical figures in teaching materials to validate identities and promote belonging [105].
4. Safe and Affirming Spaces: ITE providers should establish policies and practices that protect LGBTQ+ students and educators from discrimination and harassment [106]. Explicitly educate pre-service teachers through the ITE curriculum on how to foster safe and affirming classroom environments in their schools for the learners that they teach.
5. Intersectional Approach: ITE providers should address the overlapping identities of LGBTQ+ individuals, including race, disability, and socio-economic status, to ensure comprehensive inclusion [103]. In addition, pre-service teachers and school-based mentors should be supported to engage with Crenshaw’s [41] concept in intersectionality and consider its implications for the learners that they teach in schools.
6. Reflective Practice: ITE providers should encourage pre-service teachers to reflect on the work that they do in schools to address social justice concerns. Following immersion placements where pre-service teachers undertake LGBTQ+ projects with learners in school, ITE providers should ensure that pre-service teachers can reflect before and after these placements, individually and collectively, so that they can learn from each other’s practice.
7. In line with identity development theory, pre-service teachers should have frequent opportunities to reflect on their developing identities as socially just teachers. They should reflect on they type of teacher they want to become and their own biases, to foster empathy and inclusive teaching [107].

Implementing these best practices in teacher education programmes ensures that future educators are equipped to support LGBTQ+ students effectively. Inclusive education benefits all learners by promoting diversity, empathy, and social justice.

## 5. Conclusions

The findings of the systematic review are synthesised here in relation to the RQs (RQ1). The result from the published studies highlighted that pre-service teachers experience various forms of discrimination which results in mental ill-health. Some have agency to allow them to advance social justice within their schools (RQ2). The findings of our review suggest that there is evidence that pre-services teachers are aware that the contexts of the schools in which they work influence the extent to which they feel able to intertwine their professional and personal identities (RQ3). The findings suggest that pre-service teachers are skilled at interpreting the school climate and this affects the extent to which they can engage in queering. Although examples of pre-service teachers engaging in queering were limited across the studies that we reviewed, researchers are beginning to turn their attention to this (RQ4). The results from the reviewed studies also highlight that although there have been attempts to queer the teacher education curriculum, teacher educators also lack confidence in embedding LGBTQ+ content into teacher education programmes.

This paper makes important contributions to knowledge. First, it provides a novel framework to support ITE providers in embedding a strategic approach to LGBTQIA+ inclusion. Second, it provides an original framework to support providers in embedding LGBTQ+ inclusion into curriculum design. Third, it is the first systematic review that synthesises findings from across international contexts.

There are limitations to this study which must be acknowledged. The relatively small sample of papers reviewed limits generalisability of the findings. In addition, we reviewed papers that focused on the experiences of both pre-service teachers and qualified teachers rather than exclusively focusing on one group. We recognise that the experiences of pre-service teachers are different to the experiences of qualified teachers. Pre-service teachers have not achieved registered status, unlike qualified teachers, and therefore may need to proceed more cautiously within schools than teachers who are fully qualified. Arguably, it may be more difficult for them to disrupt cis-and hetero-normative cultures within schools due to unequal power relations which may restrict their agency. They are ‘guests’ in the school and have less power than other teachers. Engaging in acts of queering can be risky for pre-service teachers, particularly when their registration is dependent upon successfully passing the programme. In addition, we recognise that the mental health of pre-service teachers is affected by factors other than their sexual orientation or gender identities. They may be balancing work and study commitments, negotiating financial challenges and may be under pressure with examinations and coursework. Although qualified teachers may also experience adverse effects on their mental health, the factors which may contribute to this are, arguably, different to the factors which affect the mental health of pre-service teachers. Therefore, we suggest that the experiences of queer pre-service teachers should be investigated as a distinct group in future research.

We suggest that further research is needed to explore pre-service teachers’ experiences of LGBTQ+ immersion placements. In addition, the suggested frameworks that we have presented in this paper need to be piloted and evaluated by ITE providers. Finally, we found limited research on the experiences of trans/transgender pre-service teachers and we therefore suggest that this is an important research gap which should be addressed by researcher who are working within this field.

## Figures and Tables

**Table 1:** Review of initial key documents.

Overview	Russell [30] explores the experiences of Australian LGBTQ+ pre-service teachers, focusing on how their sexual identities intersect with their professional identities. Using Alsup’s [31] borderland discourse framework, the study investigates how these individuals navigate decisions around identity disclosure in school contexts. The article highlights the emotional and professional tensions faced by LGBTQ+ pre-service teachers and advocates for creating spaces where personal and professional identities can be openly discussed and integrated.
Methodological rigour	The study employs a qualitative methodology, drawing on interviews with 12 Australian LGBTQ+ pre-service teachers. This approach is appropriate for exploring complex identity negotiations and provides rich, contextual insights. The use of Alsup’s [31] borderland discourse as a theoretical lens adds depth to the analysis. However, the sample size is relatively small and limited to a specific geographic and cultural context, which may affect the generalizability of the findings.
Strengths	One of the key strengths of the study is its focus on a previously under-researched population, LGBTQ+ pre-service teachers in Australia. The use of in-depth interviews allows for a nuanced understanding of identity management and the emotional labour involved in teaching. The application of borderland discourse theory provides a robust framework for interpreting the data and underscores the importance of integrating personal identity into professional development.
Limitations	Despite its contributions, the study has several limitations. The small sample size and the focus on Australian participants limit the applicability of the findings to broader contexts. Additionally, the study does not extensively explore institutional factors such as school policies or administrative support, which could significantly impact identity negotiation. Future research could benefit from a more diverse sample and a multi-level analysis that includes institutional dynamics.
Overview	Cantos, Moliner and Sanahuja [32] conducted a descriptive study titled ‘Making sexual diversity visible through LGBTIQ+ teachers’ life stories’ published in Teaching and Teacher Education. The research aimed to explore how life stories of LGBTIQ+ teachers influence future educators’ perceptions of gender and sexual diversity. The study involved 101 students from bachelor’s and master’s programmes at Universitat Jaume I, Spain, and employed both quantitative and qualitative methods to assess changes in attitudes and awareness following exposure to these life stories.
Methodological rigour	The study utilized a mixed-methods approach, combining surveys and qualitative feedback to gather data. The use of both quantitative and qualitative data enhances the validity and depth of the findings. However, the study’s descriptive nature and limited sample size may constrain the generalizability of its conclusions. The authors did not elaborate on the training or selection criteria for the LGBTIQ+ teachers whose stories were shared, which could affect the consistency and impact of the narratives presented.
Strengths	One of the key strengths of the study is its innovative use of life stories as a pedagogical tool to foster inclusivity and awareness among trainee teachers. The findings suggest that such narratives can effectively challenge cis/heteronormative assumptions and promote empathy. The study also highlights the need for integrating gender, sexual, and family diversity (GSFD) training into teacher education curricula, which is a valuable recommendation for educational policy and practice.
Limitations	Despite its strengths, the study has several limitations. The sample was restricted to a single institution, which may limit the applicability of the results to broader contexts. Additionally, the lack of detailed information about the life stories and the absence of a control group makes it difficult to isolate the specific impact of the intervention. There is also a risk of bias in self-reported data, and the study does not address potential negative reactions or resistance among students.
Overview	Johnson [16] investigates how heteronormativity subtly persists in UK primary schools despite legislative and policy reforms aimed at promoting LGBTQ+ inclusivity. Drawing on Foucault’s concept of the panopticon, the study introduces the idea of “panoptic heteronormativity” to describe how teachers internalize and reproduce normative discourses, even while advocating for inclusive education. The research highlights the tensions between advocacy and conformity, particularly for LGBTQ+ teachers who experience a “double consciousness” in navigating their professional roles.
Methodological rigour	The study employs Interpretive Phenomenological Analysis (IPA) to explore the lived experiences of 12 UK primary school teachers engaged in LGBTQ+ advocacy. IPA is well-suited for this purpose, allowing for deep exploration of personal meaning-making. The sample size, while small, is typical for IPA studies and provides rich qualitative data. However, the study could benefit from greater demographic diversity and triangulation with other data sources (e.g., policy documents or classroom observations) to enhance validity.
Strengths	➢ Theoretical innovation: The concept of panoptic heteronormativity offers a fresh lens for understanding how power operates in educational settings. ➢ Focus on lived experience: The use of IPA provides nuanced insights into the emotional and professional complexities faced by LGBTQ+ educators. ➢ Policy relevance: The study offers actionable recommendations, such as improving teacher training and fostering critical reflection on normative discourses.
Limitations	➢ Limited generalisability: The small, homogenous sample restricts the applicability of findings across different school contexts. ➢ Potential bias: Participants were self-selected advocates, which may skew the findings toward more reflective or critical perspectives. ➢ Lack of institutional analysis: The study focuses on individual experiences but does not deeply interrogate the role of school leadership or systemic structures in perpetuating heteronormativity.
Overview	Unwin, Starcevich, Lembo and Dobson [33] explore the integration of queer perspectives into Initial Teacher Education (ITE), highlighting the challenges queer educators face in navigating professional identity within heteronormative educational contexts. The article presents four reflective vignettes drawn from the authors’ lived experiences, illustrating the complexities of becoming a queer educator. These narratives are framed by critical theory and queer theory, emphasizing the systemic forces that marginalize LGBTQIA+ identities in education.
Methodological rigour	The authors employ a qualitative, narrative methodology, using reflective vignettes to convey lived experiences. This approach aligns with Clandinin and Caine [34], who advocate for narrative inquiry as a means to understand personal and professional identity. The use of critical theory and queer theory provides a robust analytical framework, allowing the authors to interrogate heteroprofessionalism and systemic discrimination. However, the methodology is limited by its subjective nature and lack of triangulation, which may affect the generalizability of findings.
Strengths	One of the key strengths of the article is its multivocality, presenting diverse queer experiences that challenge dominant discourses in education. The integration of theory with personal narrative offers deep insight into the lived realities of queer educators. The article also provides practical recommendations for teacher educators and institutions, such as queering the curriculum and creating safe spaces for LGBTQIA+ staff.
Limitations	Despite its strengths, the article has limitations. The reliance on personal vignettes may limit its applicability across broader educational contexts. The absence of empirical data or participant voices beyond the authors restricts the scope of analysis. Additionally, while the article discusses systemic issues, it does not fully explore intersectionality or the experiences of queer educators from diverse racial or socio-economic backgrounds.

**Table 2:** Systematic steps undertaken.

Systematic Approach	Analysis of Papers [16,30,32,33]
	Inclusion and exclusion criteria
	Searching studies and literature for relevance to inclusion criteria using PRISMA
	Link to review questions
	Quality and relevance
	Synthesizing the findings
	Conclusion and recommendations

**Table 3:** Inclusion and exclusion criteria.

**Inclusion Criteria**	**Rationale**
Geographical spread	Should have international scope to reflect experiences of pre-service teachers who identify as LGBTQ+ globally.
Age-range	Literature should focus on LGBTQ+ pre-service teachers’ experiences, training.
Research base	Literature should be based on empirical research or commissioned studies (either qualitative, quantitative or mixed methods).
Date range of publications	All relevant from 2013 to 2025.
Topic	Publications must have a direct link to one of the research questions.
Reliability/validity	Literature findings must be based on valid, reliable evidence.
**Exclusion Criteria**	**Rationale**
Literature that has not been peer reviewed	To ensure robustness of research underpinning the literature.
Literature not written/published in English	Difficult to translate and related costs.
Articles published prior to 2013	Literature prior 2013 did not take account of applicable legislation.

**Table 4:** Keywords selection.

Keyword 1	Keyword 2	Keyword 3	Keyword 4
LGBTQ+	Early Career teachers	Queer	Teacher education
Pre-Service teachers	Teachers	Trainee teachers	Mental Health

**Table 5:** Keyword search combinations used.

Keyword Search Combinations Used					
(1)	(2)	(3)	(4)	(5)	(6)
LGBTQ+ AND Pre-service teachers	LGBTQ+ AND Early Career Teachers	LGBTQ+ AND Trainee Teachers	Queer AND Pre-service-teachers	LGBTQ+ AND Teacher Education	LGBTQ+ Teachers AND Mental health

**Table 6:** Database keyword search results completed on 20 July 2025.

Database/Keyword	(1)	(2)	(3)	(4)	(5)	(6)
ProQuest	335	1585	0	2938	60,195	75,307
Education Source Ultimate	56	2	4	43	274	249
ERIC	68	1	4	38	158	88
Sage Research Methods	7	7	7	7	4	3

**Table 7:** Overview of key studies included in this paper.

Reference	Overview	Research Design	Country	Setting	Sample	Data Collection Method
[30] Russell, 2021	Pre-service teachers used identity management and identity negotiation strategies.	Qualitative	Australia	ITE	Pre-service teachers	Interviews
[32] Cantos, Moliner, Sanahuja, 2023	The research recommended integrating LGBTQ+ life stories into ITE programmes.	Mixed methods	Spain	Secondary school	Pre-service teachers	Questionnaire and life story interviews
[16] Johnson, 2024	The research found that teachers who advocated for LGBTQ+ inclusivity in schools’ risk being viewed as pushing an agenda and they experience a ‘double consciousness’.	Qualitative	UK	Primary school	Teachers	Semi-structured interviews
[49] Henderson, 2019	The research found that there are conflicting obligations on LGBTQ+ teachers to both hide their identities and act as role models.	Qualitative	UK	Secondary school	Teachers	Narrative interviews
[50] Tompkins, Kearnes, & Mitton-Kükner, 2019	The study found that pre-service teachers were worried about ‘coming out’, but some felt empowered and accepted. Others felt that they were the only advocate for LGBTQ+ inclusion in their schools.	Qualitative	Canada	ITE	Teachers	Interviews
[51] Cutler, Adams & Jenkins, 2022	The research found that the willingness of pre-service teachers to transform their teaching by integrating LGBTQ+ content was influenced by their past experiences.	Mixed methods	Australia	ITE	Pre-service teachers	Survey (*n* = 38) Focus group (*n* = 4)
[52] Leent, Kay, Wighton, Peters, & Ryan, 2024	The study provides an example of a ‘queer thriving’ intervention in ITE which developed the confidence of pre-service teachers and helped them to appreciate positive narratives of LGBTQ+ lives.	Qualitative	Australia	ITE	Pre-service teachers	Workshop conversations
[53] Brant, 2017	The study found that pre-service teachers were aware of the influence of the school’s context in relation to how it affects the extent to which they can queer the school curriculum.	Mixed methods	US	ITE	Pre-service teachers	Qualitative and quantitative surveys
[54] Brant, & Willox, 2021	The study found that teacher educators were less likely to address LGBTQ+ than race.	Mixed methods	US	ITE	Teacher educators	Mixed methods survey
[55] Rhodes, Byrne & Boron, 2024	The study found that teacher educators did not feel confident about LGBTQ+ -related language/terminology and that the concept of intersectionality was poorly understood among teacher educators.	Mixed methods	Australia	ITE	Teacher Educators	Survey
[56] Mitton-Kukner, Kearns, & Tompkins, 2016	The research found that embedding LGBTQ+ content into teacher education programmes results in pre-service teachers feeling more confident. The study also found that pre-service teachers were aware of power structures in school which limit their agency.	Mixed methods	Canada	ITE	Pre-service teachers	Focus group (*n* = 6) Interviews (*n* = 6)
[57] Parker 2024	This is an auto-ethnographic account of how one beginning teacher negotiated her personal and professional identities.	Case study	US	ITE	Teachers	Interviews
[58] Brett, Bodfield, Culshaw, & Johnson, 2024	The study found that LGBTQ+ teachers were aware of power structures in schools. They self-policed their identities but were also able to reclaim some agency by disrupting heteronormative spaces.	Qualitative	UK	Teachers	Teachers	Focus group
[59] Doungphummes, & Phanthaphoommee, 2024	The study found that LGBTQ+ teachers self-regulated their personal and professional identities and that they changed their voices and gestures as a negotiation strategy.	Qualitative	Thailand	Teachers	Teachers	Interviews
[60] Millers, & Lewis, 2025	This study was included to support our understanding of school cultures from the perspective of students. Although it did not address our RQs, it provided a unique insight into the experiences of recent school graduates. The participants reported experiences of discrimination, persecution and oppressive and liberatory school environments.	Qualitative	Australia	Teachers	School graduates	Interviews
[61] Ferfolja, & Hopkins, 2013	The study found that discourses of professionalism and heteronormativity work to constrain the identities of LGBTQ+ teachers. The results also highlighted how teachers separate their private and public lives.	Qualitative	Australia	Teachers	Teachers	Semi-structured interviews and document analysis
[62] Gray, 2013	The study found that LGBTQ+ teachers were silencing their personal identities in school contexts.	Qualitative	UK	Teachers	Teachers	Life history interviews
[63] Saxey, 2021	The study found that LGBTQ+ teachers utilised their identities as queer teachers to challenge LGBTQ+ discrimination in schools.	Qualitative	English speaking countries	Primary, secondary, FE, HE	Teachers	Autobiographical accounts
[1] Gray, Harris, & Jones, 2016	The results indicated that LGBTQ+ teachers experienced stigma and occupied spaces of exclusion in schools. However, they were able to find points of interruption to advance inclusion and social justice.	Qualitative	Australia	Teachers	Teachers	Interviews
[33] Unwin, Starcevich, Lembo, & Dobson, 2024	The article presents four reflective vignettes drawn from the authors’ lived experiences, illustrating the complexities of becoming a queer educator.	Qualitative	Australia	Teachers	4 teachers	Reflective vignettes

**Table 8:** Inclusion and exclusion criteria [64].

Level/Criterion	Methodological Quality	Methodological Relevance	Topic Relevance
1: Excellent	Excellent research design with clear justification of all decisions, e.g., sample, instruments, analysis. Clear evidence of measures taken to maximize internal and external validity and reliability and reduce sources of bias.	Research questions (RQ) clearly stated. Methodology is highly relevant to their RQs and answers them in detail.	Study is very closely aligned to one of the key review objectives and provides very strong evidence upon which to base future policy/action.
2: Good	Research design clearly stated with evidence of sensible decisions taken to provide valid and reliable findings.	RQs are explicit or can be deducted from text. Findings address RQs.	Study is broadly in line with one of the key review objectives and provides useful evidence.
3: Satisfactory	Research design may be implicit but appears sensible and likely to yield useful data.	RQs implicit but appear to be broadly matched by research design and findings.	At least part of the study findings is relevant to one of the key review objectives.
4: Inadequate	Research design not stated or contains flaws.	RQs not stated or not matched by design.	Study does not address any key research objective.

**Table 9:** Frequency of papers with Weight of Evidence Rating.

	Weight of Evidence *		
	Methodological Quality	Methodological Relevance	Topic Relevance
Excellent	*n* = 0	*n* = 0	*n* = 0
Good	*n* = 10	*n* = 16	*n* = 16
Satisfactory	*n* = 9	*n* = 3	*n* = 4
Inadequate	*n* = 1	*n* = 1	*n* = 0

* Not all papers had the same rating of each criterion (overall WoE excellent in each category, *n* = 0%).

**Table 10:** Synthesizing of the findings for each research question.

Research Question	Synthesis of Findings in Summary
RQ1	Pre-service teachers experienced various forms of discrimination, but some had more positive experiences and had agency to facilitate the advancement of social justice.
RQ2	Pre-service teachers negotiated their personal and professional identities, with some making a deliberate choice to separate their personal and professional identities.
RQ3	Some pre-service teachers did not feel adequately prepared to challenge/disrupt the cultures in their schools due to their teacher education programmes not addressing LGBTQIA+ related matters.
RQ4	There are examples in the papers of deliberate efforts by teacher education providers to queer the teacher education curriculum. These include the use of guest speakers and diverse literature. Results also demonstrate that some teacher educators lacked the confidence to queer the teacher education curriculum.

**Table 11:** Search strategy.

Term	Field	Term, Synonyms and Truncation
Pre-service teacher	Title of articles and reports	(‘Pre-service teacher’ OR ‘teacher’ OR ‘Trainee teachers’ OR ‘Career teachers’)
LGBTQ+	Title, Abstract; Keywords, Journal, Author	(‘LGBTQ+’ OR ‘LGBTQ+ teachers’ OR ‘Pre-service teachers LGBTQ+’ OR ‘Trainee teacher LGBTQ+’ OR ‘Mental health’)
Mental health	Title, Abstract; Keywords, Journal, Author	‘(LGBTQ+’ OR ‘Teachers in education’ OR ‘Trainee teachers’ OR ‘Pre-service teachers’ AND ‘Mental health’)

**Table 12:** Mapping themes to the research questions.

Research Question	Theme (s)
RQ1	Discrimination; agency
RQ2	Identity
RQ3	Role models; queering
RQ4	Teacher Education

## Data Availability

All data used in this article are available on Open-Source platforms.

## Supplementary Materials

The following supporting information can be downloaded at: https://www.mdpi.com/article/10.3390/ijerph23010115/s1, PRISMA 2020 Check list.
